# Possible Role of Nutritional Priming for Early Salt and Drought Stress Responses in *Medicago truncatula*

**DOI:** 10.3389/fpls.2012.00285

**Published:** 2012-12-21

**Authors:** Christiana Staudinger, Vlora Mehmeti, Reinhard Turetschek, David Lyon, Volker Egelhofer, Stefanie Wienkoop

**Affiliations:** ^1^Department of Molecular Systems Biology, University of ViennaVienna, Austria

**Keywords:** salt stress, plant-microbe interactions, drought stress, *Medicago truncatula*, mapman mapping

## Abstract

Most legume species establish a symbiotic association with soil bacteria. The plant accommodates the differentiated rhizobia in specialized organs, the root nodules. In this environment, the microsymbiont reduces atmospheric nitrogen (N) making it available for plant metabolism. Symbiotic N-fixation is driven by the respiration of the host photosynthates and thus constitutes an additional carbon sink for the plant. Molecular phenotypes of symbiotic and non-symbiotic *Medicago truncatula* are identified. The implication of nodule symbiosis on plant abiotic stress response mechanisms is not well understood. In this study, we exposed nodulated and non-symbiotic N-fertilized plants to salt and drought conditions. We assessed the stress effects with proteomic and metabolomic methods and found a nutritionally regulated phenotypic plasticity pivotal for a differential stress adjustment strategy.

## Introduction

Reduced water availability will dramatically impact agricultural productivity in the next 40 years. According to demographic and climate change models, the human population will double by 2050 and the variability in rainfalls will increase (IPCC, 2007). Therefore, we need a profound understanding of plant physiology and metabolism under water limiting conditions.

Drought and salinity are environmental constraints accounting for substantial yield losses. Both decrease the amount of water available to plants, leading to reduced growth, and photosynthesis (Chaves et al., 2009). Thus, it has been proposed that early acclimatory responses to both stresses share strong commonalities (Munns, 2002).

Legumes play an important role in increasing the sustainability of agricultural land use. Amongst several studies on drought and salt stress effects in model legumes, many have been conducted with *Medicago* spp. recently (Lopez et al., 2008; Bianco and Defez, 2009; Salah et al., 2009; Aranjuelo et al., 2011; Filippou et al., 2011; Kang et al., 2011). Noticeably, the symbiotic status amongst the studies is very diverse. The stress response of N-fixation in root nodules was extensively studied (Larrainzar et al., 2007, 2009; Naya et al., 2007; Lopez et al., 2008; Salah et al., 2009). However, various publications have been conducted with non-symbiotic (not inoculated with rhizobia) legumes (Sanchez et al., 2008a; Noreen and Ashraf, 2009; Diaz et al., 2010). Interestingly, a positive impact of rhizobial symbiotic interaction to stress has been proposed (Frechilla et al., 2000; Miransari and Smith, 2009). However, the influence of symbiotic interactions on abiotic stress acclimatory mechanisms is still in its infancy.

During their life-cycle plants acclimate to environmental constraints by a wide range of mechanisms that are conceptually classified as avoidance or tolerance strategies (Levitt, 1980). In case of lowered water availability in the environment, stress avoidance essentially aims at maintaining the initial plant water status and lowering the rate of stress imposed at the tissue or cellular level. Tolerance strategies aim at preventing damage and maintaining metabolism, once water deficit has been established. Avoidance and tolerance mechanisms are neither mutually exclusive nor active in a temporal sequence. Their distinction is conceptual, but useful when investigating plant stress responses (Verslues et al., 2006).

Plant acclimatory responses are complex exhibiting multigenic and interrelated properties. In addition, comparability with previous work is known to be hampered, due to heterogeneities in factors influencing stress responses such as plant age, growth conditions, diurnal changes, and the experimental treatment, such as severity, duration, and method of stress imposition (Aguirrezabal et al., 2006). Consequently, robust parameters for a specific definition of stress are still missing. Due to the complexity of plant stress response and its interlinked mechanisms and influencing factors, it becomes necessary to extend research to multilevel analyses (Jogaiah et al., 2012). Using systems biology approaches the integration of -omics data such as metabolomics and proteomics may also compensate method specific limitations.

To date, data of proteomic studies are still behind in numbers of identifications that of transcript data. Nevertheless, the informative value on the protein level seems high for several reasons. For instance, the direct translation of transcript abundance to protein abundance in terms of one point abundance and changes over time is still under controversial debate. Especially, in the context of changes in time- and stress dependent manner it has been shown that transcript and protein data do not correlate significantly (Hajduch et al., 2010). As a possible reason they suggest for instance regulation via post-translational protein modification. A temporal lag that causes, e.g., a delay in adjustment of enzyme abundance when transcript levels have already changed, have extensively been discussed by Gibon et al. (2004, 2006).

So far, most studies focused on genetic engineering using, e.g., Quantitative Trait Loci (QTL) mapping have shown only limited success (Rispail et al., 2010). Thus, knowledge transfer from transcript and genome data complemented with postgenomic metabolite and proteome data will enhance the success for smart breeding in future.

In the present study, early stress response mechanisms to salt and drought stress have been investigated. The aim of this work was to (i) unravel robust and easily detectable putative stress response markers on a physiological, metabolite as well as protein level and (ii) to find novel insights for a regulatory relevant role of the nutritional priming comparing shoots of N-fixing with fertilized *M. truncatula* plants.

## Materials and Methods

### Plant growth and sampling conditions

The seeds of barrel medic (*M. truncatula* A17 cv. Jemalong) were surface sterilized and sown in pots containing a mixture of perlite:vermiculite 2:5 (v:v). The experimental setup was based on the protocol used by (Larrainzar et al., 2009). Plants were grown under controlled conditions in a growth chamber (14-h day and 10-h night; 270 μmol m^–2^ s^–1^ photosynthetic photon flux density; 22°C day and 16°C night temperatures; 50–60% relative humidity). During the first week of growth, plants were watered with nutrient solution (Evans, 1981) containing 0.5 mM ammonium nitrate. The following 2 weeks a nutrient solution with ammonium nitrate concentration of 2.5 mM was used for watering in order to enhance biomass accumulation and to keep plant growth performance identical during the initial developmental stage. After 3 weeks, half of the plants were randomly selected and inoculated with *S. meliloti* 2011. Furthermore, for inoculated plants nutrient solution was N free while the other subset was fertilized with 2.5 mM ammonium nitrate. After 7 weeks plants were randomly separated into sub-sets: control and drought or salt stressed, respectively. Control plants were supplied daily with nutrient solution to pot capacity whereas abiotic stress was applied to the other groups as follows. Drought stress was imposed by withholding water and nutrients; after flushing pots with deionized water, nutrient solutions containing 200 mM NaCl were applied every day to salt stressed plants. After 6 days of stress, plants were harvested 6 h after the onset of light. *M. truncatula* shoot and root tissue was separated, flash-frozen in liquid nitrogen, and stored at −80°C until further processing. Analysis was carried out as previously described using 3 biological replicates for each condition: N-fertilized and inoculated plants [*n* the following referred to as N-fed and nitrogen fixing (N-fix)] exposed to salt stress or water deprivation as well as control without stress treatment.

### Physiological parameters

Stomatal conductance (*g*_s_) was measured 3 h after onset of the photoperiod with a steady-state porometer (PMR-4, PP Systems, Hitchin, UK) connected to the EGM-4 gas monitor serving as data logger. About 0.5 cm^2^ of terminal leaflets of fully expanded leaves were placed into a cuvette. Records were taken after ∼20 s, when equilibrium was established. The inlet air flow rate was kept constant at 75 ml/min. The porometer then measured the air humidity of inlet and outlet air flow, air temperature and the PPFD reaching the leaf. From these parameters *g*_s_ was calculated. The water content (WC) of the leaves and roots was calculated as (FW−DW)*FW^−1^(FW = fresh weight; DW = dry weight). Leaf water potential was measured 3 h after the onset of the photoperiod with a Scholander pressure bomb. Primary chlorophyll fluorescence parameters (*F*_m′_, *F*′) were assessed employing a saturation pulse method, using the MINI-head version of the IMAGING-PAM chlorophyll fluorometer M-series (Heinz Walz GmbH, Effeltrich, Germany). The PSII operating efficiency was calculated by *F*_q_′/*F*_m′_ = (*F*_m_′−*F*′)/*F*_m_′ (Baker, 2008). Analysis was carried out on three biological replicates for each of the previously described conditions (Table 1).

**Table 1:** **Effect of drought and salt treatments on plant water status and physiological parameters in N-fed **(A)** and N-fix **(B)***M. truncatula***.

Parameter	Control	Drought	Salt
**(A) N-FED**			
WC_shoot_	0.82 ± 0.06 a	0.78 ± 0.01 b	0.82 ± 0.01 a
Ψ_leaf_ (MPa)	−0.68 ± 0.07 a	−1.06 ± 0.08 b	−0.69 ± 0.18 a
*g*_s_ [mmol m^−2^ s^−1^]	381.52 ± 139.02 a	121.52 ± 30.32 b	37.01 ± 5.46 c
*F*′_q_/*F*′_m_	0.55 ± 0.05 a	0.44 ± 0.07 b	0.58 ± 0.03 a
**(B) N-FIX**			
WC_shoot_	0.89 ± 0.01 a	0.89 ± 0.01 a	0.90 ± 0.01 a
Ψ_leaf_ (MPa)	−0.73 ± 0.10 a	−0.98 ± 0.09 b	−0.75 ± 0.17 a
*g*_s_ [mmol m^−2^ s^−1^]	425.95 ± 156.23 a	165.71 ± 36.15 b	36.14 ± 6.40 c
*F*′_q_/*F*′_m_	0.57 ± 0.02 a	0.56 ± 0.01 a	0.58 ± 0.02 a

*Values represent the mean ± SE (*n* = 3). The letters a, b, and c indicate significant differences between control and stress treatments (Student’s *t* test *p* < 0.05). WC, water content; Ψ_leaf_, leaf water potential; *g*_s_, stomatal conductance; *F*′_q_/*F*′_m_, PSII operating efficiency*.

### Extraction and derivatization of metabolites

*Medicago truncatula* roots and shoots were ground to a fine powder under liquid nitrogen and subsequently lyophilized. About 10 and 30 mg of the powdered shoots and roots were used for the extraction with 1 ml of freshly prepared and pre-cooled extraction buffer (MeOH:CHCl_3_:H_2_O, 2.5:1:0.5), respectively. In order to avoid any degradation or modification of metabolites the samples were kept on ice for 8 min. During this time the samples were vortexed regularly and afterward centrifuged for 4 min at 14,000 *g*/min, at 4°C. The supernatant was added to another tube which contained 500 μl of ultrapure water and shaken thoroughly. After the phase separation by centrifugation (4 min, 14,000 *g*/min), the upper polar phase was split into two aliquots. Internal standard (IS) was added (10 μl of 0.1 g/l^13^C_6_-Sorbitol) and the samples were dried out using a vacuum concentrator at room temperature. For metabolite derivatization, 20 μl of the freshly prepared methoximation mixture (40 g/l methoxyamine hydrochloride CH_3_ONH_2_*HCL in pyridine) were added to the dried samples and shaken for 90 min at 30°C. After adding 80 μl of the silylation mixture: 1 ml of MSTFA (*N*-methyl-*N*-trimethylsilyl trifluoroacetamide) spiked with 30 μl of the alkane standard mixture (C10-C40, each 50 mg/l) as retention index (RI) marker, the samples were incubated for 30 min with shaking at 37°C and then centrifuged (14,000 *g*/min) for 2 min to remove any insoluble material. The supernatant was carefully taken and transferred into glass vials with micro inserts. One microliter of the derivatized sample was injected. Six replicates per treatment (three biological, two technical) were randomly injected to discriminate technical from biological variation.

### GC-TSQ-MS settings

For metabolite profiling GC-MS is mostly the method of choice. Here we used GC hyphenated to triple quadrupole (Thermo Scientific TSQ Quantum GC™, Bremen, Germany). In order to identify a large number of metabolites, a profiling analysis in full-scan mode was performed with a scan range of m/z 40–600 and a scan time of 200 ms. The metabolite separation was performed on a HP-5MS capillary column (30 m × 0.25 mm × 0.25 μm; Agilent Technologies, Santa Clara, CA, USA), at a constant flow 1 ml/min helium. The split less injection of 1 μl of the sample was done by the TriPlus auto sampler (Thermo Scientific, Bremen, Germany). The temperature of the injector was 230°C. Compound elution settings were 1 min at 70°C isotherm, ramp to 76°C at 1°C per min heating rate, then to 350°C at a 6°C per min rate and hold for 1 min. Post run temperature was set to 325°C for 10 min. The transfer line temperature was set to 340°C and ion source temperature was 250°C. Electron Impact (EI) ionization was set to 70 eV.

### Metabolite detection, identification, and relative quantification

The criteria used for identification were fragmentation patterns that are characteristic for the particular compound, the retention time (RT) and RI. Combining these criteria, it is possible to unambiguously identify metabolites and distinguish between the components even if they are chemically very similar. The identification of each analyte was achieved by matching the MS-spectra and RT against (a) an in-house library (modified gmd database)^1^; (b) AMDIS (calculation of retention indices and comparison with RI of compounds in the mass spectral library); and (c) matching against the in-house measured standards. Calculation of retention indices was performed using the RT of the detected compound and the RT of the RT-index marker (alkane mixture), calculated with AMDIS for representative samples of different treatments. Due to derivatization, in some cases more than one peak was detected for one metabolite. These peaks were initially analyzed separately and summed up for further analysis or data mining. About 15% of the detected analytes were identified as unknown compounds. Calculation of the peak areas was performed using LC-Quan for the GC-TSQ-MS data, which is suitable to calculate the peak area for all compounds in all samples according to given parameters. Here the determined RT as well as the quant mass for each component was used to automatically extract data from all sample replicates. An initial data matrix of the calculated peak area for each detected compound was obtained separately. The list of detected components and calculated areas was exported to an Excel file. We used an in-house Matlab tool to produce a complete data matrix automatically. The data matrix was normalized to the sample DW and the IS for relative quantification.

### Protein extraction

The same three biological replicates as those taken for metabolite analysis have been used for protein extraction. Two hundred milligrams of liquid nitrogen frozen shoot material were cryo-ground using a Retsch MM400 ball mill and homogenized in 1 ml of urea buffer containing 50 mM HEPES, pH 7.8, 5 mM PMSF, and 8 M Urea. After centrifugation (10,000 *g*, 10 min, 4°C) the urea soluble proteins in the supernatant were precipitated overnight in five volumes of −20°C cold acetone containing 0.5% β-mercaptoethanol. The precipitate was pelleted at 4,000 *g*, 4°C for 15 min. The resulting pellet was washed with −20°C cold methanol and again centrifuged (4,000 *g*, 4°C, 10 min).

### Protein digestion

Air-dried protein pellets were dissolved in 500 μl urea buffer the protein concentration was determined by Bradford assay, using BSA as a standard. 100 μg of protein was initially digested using endoproteinase LysC (1: 100 vol/vol, 5 h, 30°C, Roche, Mannheim, Germany). For the second digestion step, samples were diluted with trypsin buffer (10% ACN, 50 mM AmBic, 2 mM CaCl_2_) to a final concentration of 2 M Urea and incubated overnight at 37°C with Porosyzme immobilized trypsin beads (1:10, vol/vol; Applied Biosystems, Darmstadt, Germany). The digest was desalted with C18-SPEC 96- well plates (Varian, Darmstadt, Germany) according to the manufacturer’s instructions. The eluted peptides were vacuum-dried.

### nanoESI LC-MS/MS

Peptide digests (0.5 μg each) were randomly applied to a RP monolithic capillary column (50 μm internal diameter, 15 cm length, Merck, Darmstadt, Germany) separated during a 120 min gradient ranging from 90% solvent A (0.1% FA in water) to 80% solvent B (80% acetonitrile, 0.1% FA in water). For each treatment tree biological and three technical replicates were randomly analyzed. MS analyses were performed on a LTQ-Orbitrap XL (Thermo Fisher Scientific, Bremen, Germany). For the database dependent spectral count analysis (Wienkoop, 2011), a top five MS analysis setting was used with the full-scan range from 350 to 1,800 m/z. Dynamic exclusion settings were as described in Hoehenwarter and Wienkoop (2010). Briefly, repeat count was set to one, repeat duration 20 s, exclusion list size 500, exclusion duration 60 s and exclusion mass width 10 ppm. Charge state screening was enabled with rejection of unassigned and 1+ charge states. Minimum signal threshold counts were set to 1,000.

### Protein identification and relative quantification

We used the SEQUEST algorithm and the Proteome Discoverer (v 1.3, Thermo Scientific) to search MS data against a fasta file we created from a *Medicago* spp. and *Sinorhizobium* spp. subset of UniProt Knowledgebase^2^ containing 63,688 sequences as of April 2012. *In silico* peptide lists were generated with the following settings: trypsin as the digestion enzyme, a maximum of three missed cleavages and methionine oxidation as dynamic modification. Mass tolerance was set to 5 ppm for precursor ions and 0.8 Da for fragment ions. Additionally, a decoy database containing reversed sequences was used to estimate the false discovery rate (FDR) Only high confidence (FDR ≤ 0.01%) peptide identifications with a minimum XCorr of 2.2 and proteins with at least two distinct peptides were considered. Peptide spectra are stored in the ProMEX library (Wienkoop et al., 2012) and can be checked under its ID “Med trun001.” Protein relative quantification is based on database dependent spectral counting as described previously (Larrainzar et al., 2009). Six replicates per treatment (three biological, two technical) were randomly injected to discriminate technical from biological variation.

### Statistical analysis

Detailed analysis of the physiology, as well as metabolite and protein data was performed by calculating the ratios between control and treated samples. Significant differences between these were determined using Student’s *t* test at *p* < 0.05 and fold change ≥ 2 (Tables 2 and 3).

**Table 2:** **Ratios of stress responsive root metabolites (stressed/control)**.

	SN-fed vs.CN-fed	SN-fix vs.CN-fix	DN-fed vs. CN-fed	DN-fix vs.CN-fix
GABA	3.1 (0.019)	0.5 (0.046)	ns	2.0 (0.043)
Aspartate	3.7 (0.011)	3.3 (0.048)	2.5 (0.049)	2.8 (0.008)
Leucine	2.0 (0.007)	ns	2.5 (0.005)	ns
Threonate	3.0 (0.046)	2.9 (0.048)	ns	2.8 (0.001)
Glutamate	4.9 (0.024)	ns	2.0 (0.001)	ns
Proline	ns	ns	10.5 (0.001)	12.1 (0.005)
Fumarate	3.5 (0.025)	2.3 (0.004)	3.1 (0.001)	3.3 (0.009)
Galactonate	3.8 (0.001)	2.8 (0.013)	ns	2.0 (0.003)
Sucrose	2.7 (0.003)	4.4 (0.006)	2.8 (0.001)	2.1 (0.003)
Myo-Inositol	4.0 (0.003)	ns	2.3 (0.002)	2.0 (0.001)
Ononitol	3.0 (0.035)	2.0 (0.003)	ns	2.0 (0.003)
Pinitol	2.6 (0.048)	ns	3.0 (0.018)	ns

*Fold change ≥ 2 and student’s *t* test *p* < 0.05 in brackets (*n* = 6). CN-fed, mean of controls of N-fertilized plant roots; SN-fed, mean of salt stressed, N-fertilized plant roots; CN-fix, mean of controls of N-fixing plant roots; DN-fix, mean of salt stressed, N-fixing plant roots; ns, not significantly changed*.

**Table 3:** **Stress responsive shoot proteins and metabolites of six replicates as fold change**.

	Stressed Drought		Stressed Salt		Non-stressed Controls
	DN-fed/CN-fed	DN-fix/CN-fix	SN-fed/CN-fed	SN-fix/CN-fix	CN-fed/CN-fix
**PROTEINS**					
**1. Photosystem (PS)**					
**1.1 PS.lightreaction**					
G7IJ45 photosystem II 10 kDa polypeptid	ns	ns	ns	3.8 (0.002)	ns
G7JH56 photosystem II CP47 chlorophyll apoprotein	ns	ns	2.2 (0.005)	ns	ns
G7JE46 thylokoid luminal 16.5 kDa protein	ns	ns	ns	0.4 (0.009)	ns
G7JAX6 photosystem I reaction center subunit N	ns	0.3 (0.029)	ns	ns	2.9 (0.043)
G7JQA7 apocytochrom *f*	0.3 (0.0029)	ns	ns	ns	ns
B7FIU4 ATP synthase gamma chain	ns	2.8 (0.003)	ns	ns	0.5 (0.010)
B7FIR4 ATP synthase gamma chain	ns	0.5 (0.009)	ns	ns	ns
G7JAI2 ATP synthase	ns	ns	3.5 (0.0030)	ns	ns
**1.2 PS.photorespiration**					
G7JAR7 serin hydroxymethyltransferase	ns	0.5 (0.019)	ns	ns	ns
**1.3 PS.calvin cycle**					
G7J252 ribulose bisphosphate carboxylase small chain	ns	ns	3.3 (0.020)	ns	3.2 (0.005)
**2.1.2 Major CHO metabolism.synthesis.starch.transporter**					
G7LDP4 ADP, ATP carrier protein	ns	ns	2.1 (0.014)	ns	ns
**3.4 Minor CHO metabolism.myo-inositol**					
G7J4B5 l-myo-inositol-1 phosphate synthase	5.3 (0.0001)	ns	ns	ns	4.0 (0.033)
G7LAD5 l-myo-inositol-1 phosphate synthase	2.0 (0.0204)	ns	ns	ns	ns
**6.3 Gluconeogenesis.Malate DH**					
G7JTZ0 Malate dehydrogenase	ns	ns	ns	0.4 (0.000)	ns
**7.1 OPP.oxidative PP.6-phosphogluconate dehydrogenase**					
Q2HVD9 6-phosphogluconate dehydrogenase	ns	0.5 (0.001)	ns	ns	2.0 (0.001)
**9.9 Mitochondrial electron transport/ATP synthesis.F1-ATPase**					
G7LCJ4 ATP synthase delta subunit	2.5 (0.0001)	ns	ns	ns	2.2 (0.048)
**10.1 Cell wall.precursor synthesis**					
G7L571 UDP-glucose 6-dehydrogenase	ns	0.3 (0.001)	ns	ns	ns
**11.1 Lipid metabolism.FA synthesis and FA elongation**					
G7LIV6 biotin carboxylase	ns	0.4 (0.032)	ns	ns	2.6 (0.023)
G7JNN1 Acyl-[acyl-carrier-protein] desaturase	ns	ns	3.6 (0.014)	ns	ns
**11.6 Lipid metabolism.lipid-transfer proteins**					
G7JID0 non-specific lipid-transfer protein	2.5 (0.002)	ns	ns	ns	ns
**12.2 *N*-metabolism.ammonia metabolism.glutamate synthase**					
Q2HW53 ferredoxin-dependent glutamate synthase	ns	0.5 (0.000)	ns	ns	ns
P04078 glutamine synthetase cytosolic isozyme	ns	ns	ns	0.5 (0.004)	3.8 (0.004)
**13.1 Amino acid metabolism.synthesis**					
Q6J9 × 6 SAMS	2.2 (0.0079)	ns	ns	ns	ns
A4ULF8 SAMS	2.4 (0.0007)	ns	ns	ns	ns
A4PU48 SAMS	ns	0.5 (0.009)	ns	ns	ns
G7L3W1 SAMS	ns	0.5 (0.002)	ns	ns	ns
G7JTY4 LL-diaminopimelate aminotransferase	ns	ns	0.4 (0.021)	ns	ns
G7J013 alanine glyoxylate aminotransferase	ns	ns	ns	2.4 (0.005)	ns
**15.2 Metal handling.binding, chelation, and storage**					
G7K283 ferritin	ns	ns	4.0 (0.018)	ns	ns
G7JLS7 ferritin	11.4 (0.005)	ns	10.0 (0.0004)	ns	ns
**16.2 Secondary metabolism.phenylpropanoids**					
G7JTH6 caffeic acid 3-*O*-methyltransferase	6.5 (0.0000)	ns	4.0 (0.0023)	ns	ns
**19.10 Tetrapyrrole synthesis**					
G7IK85 Mg-chelatase subunit chlI	0.3 (0.0002)	0.3 (0.0005)	ns	0.5 (0.004)	ns
**20.1 Stress.biotic**					
B0RZH7 putative thaumatin-like protein	ns	0.4 (0.000)	ns	ns	2.2 (0.001)
G7IYL0 receptor-like protein kinase	ns	0.5 (0.002)	ns	ns	ns
**20.2 Stress.abiotic**					
Q2HT97 heat shock protein Hsp70	ns	ns	ns	0.5 (0.034)	ns
G7JGC6 low-temperature inducible	2.3 (0.0001)	ns	ns	ns	2.3 (0.001)
G7JGC9 low-temperature inducible	ns	ns	ns	0.3 (0.021)	ns
**21.5 Redox.peroxiredoxin**					
G7JS60 peroxiredoxin Q	2.6 (0.0000)	ns	ns	ns	ns
**23.4 Nucleotide metabolism.phosphotransfer and pyrophosphatases**					
G7JMM2 nucleoside diphosphate kinase	ns	ns	2.0 (0.040)	ns	ns
B7FIM7 soluble inorganic pyrophosphatase	ns	03 (0.006)	ns	ns	ns
**26.20 Misc.ferredoxin-like**					
G7KWY5 ferredoxin	ns	0.3 (0.011)	ns	ns	2.1 (0.037)
**26.4 Misc.beta-1,3 glucan hydrolases**					
G7JQL4 endo-beta-1,3-glucanase	ns	ns	0.5 (0.014)	ns	ns
**27.1 RNA.processing**					
G7JK09 Poly(A)-binding protein	ns	0.4 (0.000)	ns	ns	ns
**27.4 RNA.RNA binding**					
G7JG67 glycerine-rich RNA binding protein	0.5 (0.0059)	ns	ns	ns	ns
**29.2 Protein.synthesis**					
Q945F4 eukaryotic translation initiation factor 5A-2	ns	0.4 (0.003)	ns	ns	2.5(0.003)
G7IH13 elongation factor EF-2	ns	ns	ns	2.5 (0.000)	ns
**29.5 Protein.degradation**					
G7LIT0 ATP-dependent Clp protease	0.4 (0.0226)	ns	ns	ns	ns
G7ZVC0 presequence protease	ns	0.5 (0.010)	ns	ns	ns
G7K8J5 bi-ubiquitin	ns	ns	0.3 (0.024)	ns	ns
G7LB82 proteasome subunit alpha type	ns	ns	2.1 (0.019)	ns	ns
**31.1 ′Cell.organization**					
G7IAN2 tubulin ß chain	ns	ns	5.4 (0.0006)	ns	ns
G7L5V0 tubulin ß chain	ns	ns	3.0 (0.0205)	0.4 (0.046)	ns
G7KB73 annexin	2.0 (0.0394)	ns	ns	ns	0.5 (0.000)
G7JAX5 actin	ns	ns	3.8 (0.0001)	ns	ns
**34.1 Transport. p- and v-ATPases**					
A6Y950 Vacuolar H + -ATPase B subunit	ns	0.5 (0.001)	ns	ns	ns
**“PUTATIVE” UNCHARACTERIZED PROTEINS**					
B7FJY9 similar 94.0% Q9SQL2, CB24_PEA, chlorophyll a-b binding protein P4, chloropl., *Pisum sativum* (garden pea), *e* = 1.0 × 10^−178^	3.0 (0.0001)	ns	ns	ns	ns
B7FMC4 similar 73.0% Q03666, GSTX4_TOBAC, probable glutathione *S*-transferase, *Nicotiana tabacum* (common tobacco), *e* = 1.0 × 10^−121^	2.1 (0.007)	ns	ns	ns	2.6 (0.036)
B7FJR8 similar 83.0% Q9LZG0, ADK2_ARATH, adenosine kinase 2, *Arabidopsis thaliana* (mouse-ear cress), *e* = 0	ns	0.4 (0.000)	ns	ns	2.1 (0.015)
B7FM78 similar 97.0% P81406, GAPN_PEA, NADP-dependent glyceraldehyde-3-phosphate dehydrogenase, *Pisum sativum* (garden pea), *e* = 0	ns	0.5 (0.023)	ns	ns	ns
B7FKR5 similar 99.0% O24076, GBLP_MEDSA, guanine nucleotide-binding protein subunit beta, *Medicago sativa* (alfalfa), *e* = 0	ns	0.4 (0.005)	ns	ns	2.6 (0.001)
B7FI14 similar 64.0% Q9LEH3, PER15_IPOBA, peroxidase 15, *Ipomea batatas* (sweet potato) (*Convolvulus batatas*), *e* = 1.0 × 10^−132^	ns	0.4 (0.000)	ns	ns	2.2 (0.001)
B7FL15 similar 85.0% P13443, DHGY_CUCSA, glycerate dehydrogenase, *Cucumis sativus* (cucumber), *e* = 2.0 × 10^−71^	ns	0.3 (0.000)	ns	ns	2.7 (0.000)
G7I4F9 uncharacterized protein	ns	0.4 (0.021)	ns	ns	2.1 (0.035)
B7FHX0 similar 98.0% P29500, TBB1_PEA, tubulin beta-1 chain, *Pisum sativum* (garden pea), *e* = 0	ns	ns	4.4 (0.0035)	ns	2.2 (0.002)
B7ZWQ5 similar 90.0% Q40977, MDAR_PEA, monodehydroascorbate reductase, *Pisum sativum* (garden pea), *e* = 0	ns	ns	2.0 (0.035)	ns	ns
B7FL16 similar 84.0% P13443, DHGY_CUCSA, glycerate dehydrogenase, *Cucumis sativus* (Cucumber), *e* = 2.0 × 10^−88^	ns	ns	ns	0.5 (0.043)	ns
B7FI41 similar 52.0% Q41160, LCB3_ROBPS, putative bark agglutinin LECRPA3, *Robinia pseudoacacia* (BLAQCK locust), *e* = 5.0 × 10^−87^	ns	ns	ns	0.4 (0.023)	ns
G7KAG7 similar 71.0% Q9THX6, TL29_SOLLC, thylakoid lumenal 29 kDa protein, chloroplast, *Solanum lycopersicum* (tomato; *Lycopersicon esculentum*), *e* = 1.0 × 10^−172^	ns	ns	ns	0.5 (0.032)	ns
B7FNH1 similar 79.0% O23755, EF2_BETVU, elongation factor 2, Beta vulgaris (sugar beet), *e* = 2.0 × 10^−67^	ns	ns	ns	0.3 (0.004)	3.6 (0.004)
**METABOLITES**					
**Major CHO metabolism **					
Glucose	ns	10 (0.034)	ns	0.3 (0.009)	0.5 (0.014)
Glucose-1-p	ns	ns	ns	5.1 (0.000)	0.5 (0.014)
Maltose	ns	ns	ns	2.3 (0.003)	ns
Ribitol	3.2 (0.010)	ns	ns	ns	ns
**Amino acid metabolism**					
Glutamate	ns	ns	2.1 (0.010)	ns	ns
Leucine	ns	6.1 (0.000)	2.7 (0.006)	2.2 (0.012)	ns
Proline	0.5 (0.049)	ns	2.6 (0.006)	ns	ns
Valine	ns	ns	2.4 (0.000)	2.4 (0.012)	ns
Aspartate	ns	0.3 (0.021)	ns	ns	ns
**TCA**					
2-Oxoglutarate	ns	ns	ns	0.3 (0.040)	ns
Citrate	ns	ns	0.5 (0.008)	0.3 (0.000)	ns
Succinate	ns	ns	ns	0.5 (0.008)	ns
Malate	ns	ns	ns	0.2 (0.001)	2.0 (0.012)
Malonate	ns	0.5 (0.029)	0.4 (0.000)	0.1 (0.001)	2.1 (0.001)
**Others**					
Phosphate	ns	ns	0.5 (0.006)	0.2 (0.000)	ns

*(Student’s *t* test *p* < 0.05 in brackets and fold change ≥ 2; *n* = 6) with significantly altered abundances in response to spectral counts of stress proteins and peak area of metabolites (IS and DW normalized). Protein category headers including binning numbers of the MapMan mapping file. CN-fed, control, N-fertilized; CN-fix, control, N-fixation; DN-fed, drought, N-fertilized; DN-fix, drought, N-fixation; SN-fed, salt, N-fertilized; SN-fix, salt, N-fixation; numbers 1 – 6 indicate replicates. ns, not significant; SAMS, *S*-adenosylmethionone synthetase*.

### Mapman mapping file for *M. truncatula* proteins and metabolites

A new Mapman mapping file was created on the basis of the mapping file “Mt_Mt3.5_0411” and “MappingMetabolites” acquired from http://mapman.gabipd.org/web/guest/mapmanstore. This mapping file corresponds to MTGI release “Mt3.5v3 RELEASE 20100825” (“Mt3.5_GenesProteinSeq_20100825.fasta” subsequently called MTGI-fasta-DB) which can be found at http://www.jcvi.org/. Shotgun proteomics experimental data were evaluated with the Uniprot database fasta file (see Protein Identification and Relative Quantification).

The “Identifier” and “Description” categories of entries from the “Mt_Mt3.5_0411” mapping file correspond to accession numbers and header information of the MTGI-fasta-DB. The mapping file ”Med trun_mappingformapman_Mosys_v1_20120913.txt” was created by comparing the protein sequences of the Uniprot-fasta-DB (MT only) to the MTGI-fasta-DB. Comparison was performed using string comparison (unpublished Python script) as well as standalone BLAST from http://blast.ncbi.nlm.nih.gov. Mapping file entries corresponding to completely identical sequences were replaced. The “Bincode” and “Name” remained unchanged, but the “Identifier” and the “Description” were replaced by the corresponding Uniprot accession number and header, furthermore the “Type” was set to “*P*.” All Uniprot entries not 100% identical in sequence and length to an entry in MTGI were blasted against a database created from the entire MTGI fasta file. Uniprot entry hits with an *e*-value equal to or lower than10^−3^ replaced mapping file entries as previously described. Uniprot entry hits with *e*-values higher than 10^−3^ were added to the bincode “35.2.1” with the name “not assigned.unknown.evalhigh” and entry hits resulting in no query hit at all were assigned to the bincode “35.2.2” with the description “not assigned.unknown.blastwithouthits.” The pertinent information of the metabolite mapping file from the MapMan Store was incorporated into the current mapping file by simply adding the respective entries (at the proper bin location). Certain entries were manually curated and shifted from “not assigned.unknown” bins to appropriate categories. Six metabolites not previously contained in the mapping were added. Separate bins were created for *S. meliloti* and *S. medicae*. Identified and pertinent protein accessions of these two endosymbionts were manually classified and thus put into sub-bins. The mapping file can be downloaded at http://www.univie.ac.at/mosys/databases.html. It will be updated in accordance to novel identifications/insights.

### Functional characterization of stress responsive putative uncharacterized proteins

For a functional characterization of the stress responsive, so far putative proteins of unknown function in our analysis, we have used BLAST to find entries in phylogenetically related organisms by sequence similarity (see also Table 3).

## Results

### Physiological responses to salt and drought in *M. truncatula*

*Medicago truncatula* was chosen in order to study the early stress acclimation under two N-nutritional conditions combined with two different environmental perturbations (four different stress treatments). The effect of reduced water availability on plant performance was analyzed in order to assess the degree of stress as alterations in water status in both nutritional phenotypes in *M. truncatula* (N-fed and N-fix; Table 1). The effect of drought stress was significant for most of the analyzed parameters depending on nutritional status. Water potential was significantly reduced during drought (potential dropped to −0.98 MPa and −1.06 MPa for N-fix and N-fed plants respectively), but not during salt stress. The PSII operating efficiency in terms of (*F*′q/*F*′m) was significantly decreased only in the leaves of drought stressed N-fed plants. Stomatal conductance was significantly reduced upon perturbation. In order to get a more holistic insight into the extent of plant acclimatory responses, significantly changing root metabolites were assessed (Table 2). Most of the significantly changed metabolites in roots did not change significantly in the leaves and vice versa. However, the degree of stress in terms of the fold change was more significant in the roots. Most of the responsive metabolites increased during stress. However, especially organic acids and a few amino acids of the leaves showed a decline in response to stress (Table 3).

### Descriptive analysis of the detected proteins and metabolites

**Figure 1:**
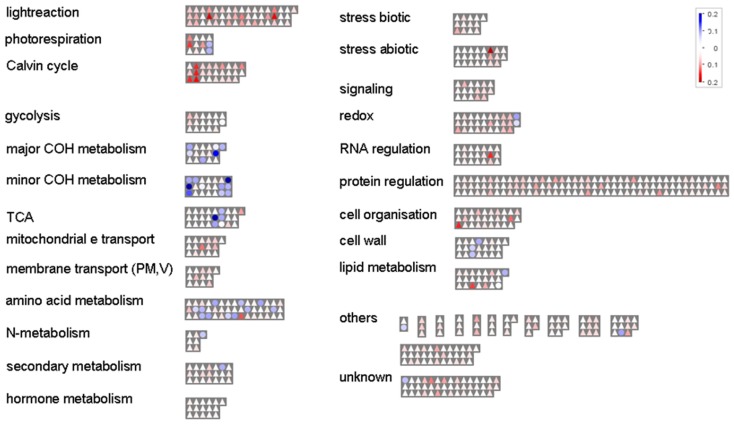
**Mapman overview**. Functional distribution and relative abundance of the 643 shoot proteins (Log10 of the spectral count normalized by protein weight; FDR ≤ 0.01%) and 45 metabolites (Log10 of peak area normalized by DW and IS) identified. Triangles = proteins; circles = metabolites. The strength of the color indicates the abundance of the compounds.

All identified proteins and metabolites were functionally categorized and visualized with Mapman (Thimm et al., 2004) using a new *M. truncatula* mapping file we created for UniProt data (Figure 1, see Mapman Mapping File for *M. truncatula* Proteins and Metabolites). Upon all identified proteins (643), “protein regulation” (20%), and “PS” (13%) are the dominant functional categories. In addition, the proteins assigned to the PS show highest relative abundance (spectral count per protein weight). Other categories are “redox,” “amino acid,” and “cell,” each accounting for 5% of all identified proteins. Stress and signaling together reach 7% of all protein identifications followed by several other small categories (Figure 1). For the metabolites we found the major categories of the primary metabolism including amino acids “AA metabolism,” the “TCA” cycle (organic acids), sugars “major COH metabolism,” and “others.”

### Quantitative data mining for salt and drought responsive metabolites and proteins of nutritional *M. truncatula* phenotypes

**Figure 2:**
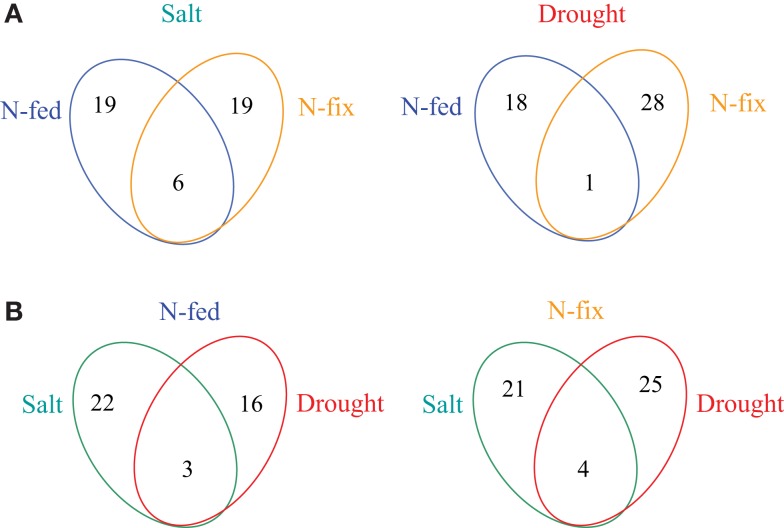
**Venn diagrams of the number of stress responsive proteins and metabolites**. **(A)** overview of N-source depended changes for drought and salt separately; **(B)** overview of stress dependent overlap for N-fed (nitrogen fertilization) and N-fix (nitrogen fixation) separately.

**Figure 3:**
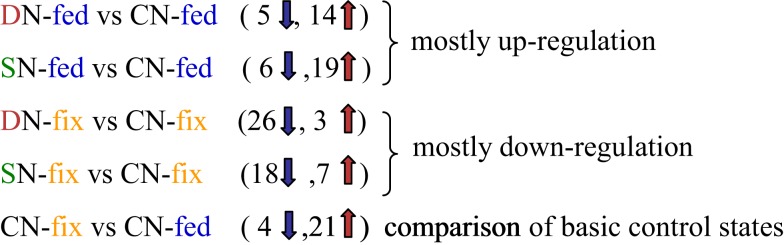
**Summary of the number of up- or down-regulated shoot compounds (proteins and metabolites) of the different treatments**. Compounds, mostly up-regulated in N-fed and mostly down-regulated in N-fix plants during stress (independent on stress condition). D, drought; C, control; S, salt; N-fed, nitrogen fertilization; N-fix, nitrogen fixation.

**Figure 4:**
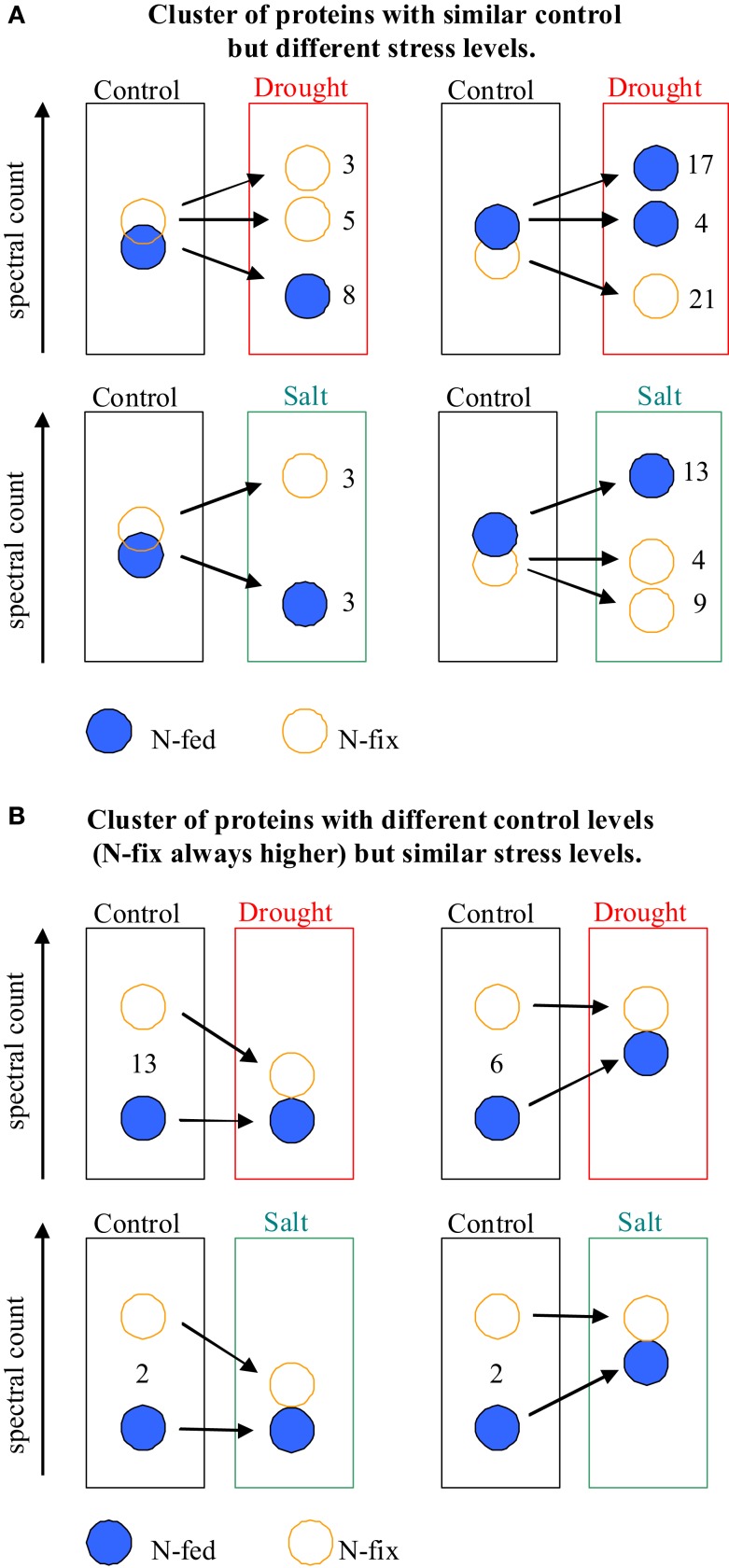
**Schematic overview of differentially up or down-regulated protein clusters depending on control level and nutritional status**. **(A)** Similar control levels but different stress levels. **(B)** Different control levels but similar stress response levels. N-fed, nitrogen fertilization; N-fix, nitrogen fixation.

About 11% of all identified proteins (69 of 643) and 33% of all identified metabolites (15 of 45) changed significantly upon early stress acclimation (*p* ≤ 0.05 and fold change ≥ 2; *n* = 6). GC-MS based metabolite profiling generally results in the identification of metabolites associated with the primary metabolism. Here, we found that most metabolites responding to stress were corresponding to the major sugar and amino acid metabolism and the TCA cycle. The protein categories with the highest percentage involved in stress response are: “PS,” “amino acid,” and “cell” with 12% each (Table 3). A small overlap of responsive compounds across the two stress treatments was observed (7 of 98, Figure 2). However, no analyzed compound was responsive during stress acclimation across all treatments. The Mg-chelatase subunit chlI (G7IK85), leucine, and malonate have been found to respond to three of the four different treatments. Of all the significantly altered levels of proteins and metabolites, only a particular subset responded to a specific treatment. Approaching the data from a different perspective, Figure 2A shows that more responsive compounds are shared between the salt than between the drought treated phenotypes. In contrast, a few specific response features were observed when dissecting the nutritional phenotypes (Figure 2B). Altogether, we found that the majority of significantly changed compounds of the nitrogen fertilized (N-fed) plants increased while the majority of significantly changed compounds of the N-fix plants decreased independent of the stress type (Figures 3 and 4).

We then compared the control levels of the proteins of the nutritional phenotypes with the response levels of perturbation (Figure 4). Interestingly, for the drought stressed plants, an approximation in protein levels between the two phenotypes has been observed. Thirteen responsive proteins of the N-fix plants show a higher control level compared to the N-fed controls. At the analyzed time of drought acclimation, those proteins decreased significantly, reaching the level of the N-fed plants (which have not changed during drought stress). Vice versa, control levels of six responsive proteins of the N-fed plants increased during drought, reaching unchanged control levels of the N-fix plants. This mechanism is less distinct for salt stress (Figure 4).

## Discussion

### Definition of the degree of stress and the challenge of comparing different constraints

Salt and drought, two major environmental constraints have been compared. A moderate stress level was applied in order to study the early acclimation responses of *M. truncatula* growing under two different nutritional conditions. A biphasic growth inhibition model by saline conditions has been proposed earlier (Munns, 2002). During the first phase, growth inhibition is mainly governed by the decreased water availability due to higher solute concentrations in the soil solution, lowering soil water potential. If salt stress is prolonged ion toxicity effects gain importance in constraining plant metabolism and survival, described as the second phase, the salt stress specific phase (Sanchez et al., 2008a). To obtain similar early stress response levels for both stress types, keeping morphological parameters comparable, plants were harvested at the same age (duration-of-stress was 6 days). We compared the response of *M. truncatula* to water deficit resulting from a progressive mild drought treatment and a high initial 200 mM salt treatment. After 6 days of treatment, water-withholding and salt stress treatment resulted in stress responses. In order to assess the degree of stress at the plant level, several physiological parameters showing typical responses to decreasing water availability were analyzed as direct and indirect measures of plant water status (Table 1). All four stress treatments elicited acclimatory responses, as evidenced by significant decreases in stomatal conductance. Furthermore, our data also indicate that stress treatments had a low effect on photosynthesis. The PSII operating efficiency was neither affected by salt nor severely by drought (Table 1). This supports the onset of an early phase of stress acclimation. Drought experiments of soybean have shown that rates of photosynthesis were inhibited when leaf water potential dropped below −1.1 MPa (Boyer, 1970). This is consistent with our data; since photosynthesis was only affected in drought treated N-fed plants, when leaf water potential reached threshold. Salt and drought constraints are initially encountered at the root part of the plants. This might also contribute to the fact that in legumes, N-fixation is impaired in response to water deficit, before a decrease in photosynthetic rate can be observed (Durand et al., 1987; Djekoun and Planchon, 1991). As expected, when testing for some significant changes of metabolites in roots compared to the shoots (Table 2), the extent of stress-induced was more important in roots than in shoots. Some typical stress response marker such as proline, GABA and the polyols pinitol, ononitol, and myo-inositol (Vernon and Bohnert, 1992) were partially found to solely or more distinctly accumulate in the roots. Surprisingly, proline was only significantly increased in roots (∼10-fold) exposed to drought and shoots (∼twofold) exposed to salt. In leaves of N-fed plants it was found to even decrease. Since proline has been reported to increase during drought (Delauney and Verma, 1993) and other abiotic effects (Szabados and Savouré, 2010), the data suggest a moderate stress response where proline accumulation has not been fully established. This observation could result from the more pronounced stomatal closure in salt stressed than in drought stressed plants. As the water loss through stomata is lower, tissue WC, and water potential remain constant. Thus the degree of stress at the plant tissue level might not yet induce a substantial accumulation of osmoprotectants such as proline. The results for drought are in agreement with the data of Filippou et al. (2011), where, e.g., proline accumulation in *M. truncatula* leaves occurred only after 9 days of water-withholding, whereas in roots already after 3 days. The biological role of proline accumulation during stress is under extensive discussion (Verbruggen and Hermans, 2008). Drought stress experiments in *Lotus japonicus* strengthened the hypothesis that proline is necessary for the rehydration ability of the plants (Diaz et al., 2010). In agreement with our data, they showed that it does not reduce the rate of water loss. Interestingly, the counter-correlation of salt stressed plants showing no changes in leaf water potential suggests that this might be due to the more significant decrease in stomatal conductance, regulated by an increased ABA level. It was shown that the stomatal conductance was controlled by the root water potential when the ABA level of the xylem sap was increased (Tardieu et al., 1991). Thus our data demonstrate that salt and drought have impact on stomatal conductance but to a different degree, indicating higher stress response to salt than to drought. In contrast, water potential decreased significantly only during drought and more severely in N-fed compared to N-fix plants leading to the conclusion that drought stress has a stronger and thus earlier impact on water availability than salt. However, effects are still in a moderate range revealing an early stress response for both constraints. Altogether the physiological results lead to the following conclusions: (a) Indifferent to stress treatment and nutritional status stomatal conductance is an early stress response parameter; (b) Proline and the other observed, typical stress responsive metabolites as well as photosynthetic efficiency seem to be robust markers only for severe stress in leaves; (c) the root is the first place adjusting and controlling acclimation of stress; (d) all physiological parameters showing significant differences when comparing control to stressed groups, interestingly also showing significant differences between the two stress treatments; (e) in order to establish the highest possible similarity in plant water status between the two constraints, numerous salt concentrations and time points need to be assessed (and possibly additional parameters measured). However, an identical response seems very unlikely.

### Molecular stress adjustments depending on the nutritional phenotype – chaos with system?

Numerous studies on salt and/or drought stress in plants have been summarized recently (Pinheiro and Chaves, 2011; Krasensky and Jonak, 2012). Drought and salinity reduce soil water availability and induce common stress avoidance strategies such as shoot growth inhibition and lower stomatal conductance. However, there is not much overlap between the molecular data sets published so far. This is probably due to the fact that experimental setups and application of stresses are very different and an appropriate definition of the degree of stress (in terms of experimental conditions as well as plant water status) for a better comparison is difficult and often missing (Jones, 2007). Another reason may be the differential steady-state of the plants such as growth state (Chaves et al., 2009) and nutritional status prior to stress exposure (Frechilla et al., 2000). The molecular data presented here shows that salt and drought stress share few common features in terms of changes in compound abundance (Table 3 and Figures 2A,B). First of all, the significantly responding compounds appear randomly distributed across treatments and most functional categories of the metabolic network. This result is not surprising since stress effects seem not severe and plant metabolism has not yet been fully adjusted at the time of analyses. However, in agreement with other data (Sanchez et al., 2008a,b), we found a down-regulation of organic acids and an up-regulation of amino acids that seem typical for salt stress (Table 3). The results suggest that the TCA cycle is almost exclusively responding to early salt stress but not to drought. Within the N-fix phenotype of the salt stress group all five of the responsive metabolites of the TCA cycle were down-regulated.

Amino acids most significantly change in salt stressed and N-fed plants while most of the responsive sugars significantly changed in N-fix plants. The protein levels of the functional categories of amino acid and N-metabolism decreased, while the amino acids accumulated in response to stress. This trend could be observed within all stress treatments, except for drought stressed N-fed plants where this trend was inverted (Table 3). Possibly, increased amino acid levels are the cause for the down-regulation of proteins involved in amino acid synthesis and/or the consequence of protein degradation. Interestingly, this correlation has also previously been observed in root nodules of drought stressed *M. truncatula* (Larrainzar et al., 2009). They also found some glutamine sythetase isoforms decreasing during drought. However, while amino acid synthetases and asparagine aminotransferases seemed to play an important role during drought stress acclimation in nodules, *S*-adenosyl-l-methionine synthases (SAMS) seem to be more specifically involved in leaves. In addition, the SAMS isoforms seem only involved in early response to drought but not to salt stress. Furthermore, the four identified SAMS isoforms respond differently to drought. SAMS is a key enzyme, catalyzing the biosynthesis of SAM using methionine and ATP. It has been described that some of the SAMS genes were expressed constitutively, whereas others seemed specifically regulated by developmental and/or environmental factors depending on the requirement for SAM (Boerjan et al., 1994; Gómez-Gómez and Carrasco, 1998). SAM is a methyl donor, involved in many regulatory relevant processes on the transcript and protein level (Gómez-Gómez and Carrasco, 1998). However, further studies need to be conducted to unravel the regulatory function of the different SAMS isoforms during plant responses to water deficits.

Sugars are usually described to increase during osmotic stress adjustment (Clifford et al., 1998; Hummel et al., 2010). Surprisingly, glucose was decreasing in salt stressed plants. However, under drought stress glucose increased and other carbon metabolites increased as well. Interestingly, on the protein level, cell organization seemed most responsive in salt stressed N-fed plants. Distinctively, the two tubulin β chains (G7IAN2 and G7L5V0) and actin (G7JAX5) were found to be up-regulated. These components are involved in the dynamics of the cytoskeleton. Several studies in Arabidopsis have shown a relationship between the plant cytoskeleton and salt stress tolerance by the induction of actin filament assembly and bundle formation (Wang et al., 2010, 2011). This result may indicate a more specific response of salt stressed plants that are N-fertilized.

Besides malonate (down-regulation) and leucine (up-regulation), the metabolites found to respond in three out of the four treatments, Mg-chelatase subunit chlI (G7IK85) was also significantly changed (down-regulated) in both drought phenotypes as well as the salt stressed N-fix plants. The Mg-chelatase, composed of three different subunits, is the first enzyme involved in chlorophyll biosynthesis. It has been described to be involved in several stress-induced alterations. Dalal and Tripathy (2012) summarized the stress response of enzyme activity and on the protein and transcript level. They showed that Mg-chelatase protein abundance and gene expression are generally down-regulated during drought, salt, cold, and heat stress. A study on pea revealed that the Mg-chelatase chlI activity is redox regulated by chloroplast thioredoxins (Luo et al., 2012). Intriguingly, there are controversial discussions dealing with the Mg-chelatase subunit chlH. Initially it has been reported to act as an ABA receptor (Shen et al., 2006). However, Müller and Hansson (2009) reported that ABA had no effect on subunit chlH. Recently, Tsuzuki et al. (2011) presented evidence for the chlH subunit affecting ABA signaling of stomata guard cells but not acting as ABA receptor. These data strongly support that the Mg-chelatase is an important key player of chlorophyll degradation already during early stress response. The role of subunit chlI, however, needs to be studied in more detail.

Most other stress responsive compounds found, appear to be selectively distributed. However, we found interesting response patterns that might be explained by regulatory important mechanism: noticeably, the ratio between up and down-regulated compounds is grouping the nutritional phenotypes (Figure 3). The different molecular control levels of the two nutritional traits are leading to these response patterns. Starting with the comparison of the phenotypes, we found 25 of the stress responsive compounds also significantly distinguish N-fix from N-fed plants under control condition (Table 3). Here in general, protein and metabolite levels are higher in the control steady states of the N-fix plants compared to the N-fed plants. Furthermore, the ratio of up- vs. down-regulated proteins and metabolites during early stress response is generally higher in N-fed plants and vice versa the ratio of down-regulation higher in N-fed plants. Several distinct proteins seemed to change randomly coming from the same control state (Figure 4A). However, when analyzing the phenotypes after early stress adjustment, the proteomic data revealed a process of approximation to a similar molecular stress-steady-state (Figure 4B). Especially the protein response-pattern to drought aligned the way that proteins of the N-fix shoots of higher control level decreased to the level of N-fed shoots and vice versa. Taking these data together, there is evidence that the N-fed plants invest more energy in stress adjustment of protein levels than the N-fixing plants, where down-regulation of proteins is dominating the process of acclimation. Interestingly, there is an overlap of six for the salt- compared to one stress responsive protein of the drought treatment (Figure 2A). Thus, salt stress response seems less dependent on the nutritional status than drought. Thus, we propose that (a) the initial molecular steady-state of the plants in terms of nutritional status seems pivotal for the downstream stress adjustment strategy; (b) during stress-acclimation-phase plants try to adjust their metabolic network to an approximate level (more significantly during the drought stress response); and (c) N-fix plants may need less energy for the stress adjustment than N-fed *M*. *truncatula* plants.

## Conclusion

In the case of *M. truncatula*, our results suggest the following.

- Our drought stress treatment, led to a more pronounced water deficiency at the plant level than the salt stress treatment. This finding points to stress type specific acclimation strategies, especially stress avoidance mechanisms such as stomatal conductance. Either way, physiological, metabolomic, and proteomic data revealed significant differences in the degree and strategy of early drought, as compared to salt stress response, under identical growth conditions.
- Mg-chelatase subunit chlI, leucin, and malonate were significantly affected in three out of four stress treatments (two stress types, two nutritional conditions). Thus, they are likely robust early stress response markers. Further evaluation studies are necessary for confirmation.
- Proteomic adjustment seems low cost for N-fixing, as compared to N-fertilized plants, suggesting a potentially increased tolerance to stress. Whether this can be explained by symbiotic interaction itself or a more general kind of nutritional priming remains to be investigated further. However our results underline that the N-nutritional condition seems of crucial importance for plant stress acclimation.

## Conflict of Interest Statement

The authors declare that the research was conducted in the absence of any commercial or financial relationships that could be construed as a potential conflict of interest.

## Footnotes

^1^http://gmd.mpimp-golm.mpg.de/download/

^2^http://www.uniprot.org/

## Abbreviations

C, control; D, drought; DW, dry weight; *F*′, chlorophyll fluorescence in the light-adapted state; FDR, false discovery rate; *F*_m′_, chlorophyll fluorescence when PSII centers are maximally closed in the light-adapted state; *F*_q′_, difference between *F*′and *F*_m′_; FW, fresh weight; *g*_s_, stomatal conductance; IS, internal standard; N, nitrogen; N-fed, nitrogen fertilization; N-fix, nitrogen fixation; PS, photosynthesis; PSII, photosystem II; S, salt; SE, standard error; WC, water content; Ψ_leaf_, leaf water potential.
